# Yoga Poses Increase Subjective Energy and State Self-Esteem in Comparison to ‘Power Poses’

**DOI:** 10.3389/fpsyg.2017.00752

**Published:** 2017-05-11

**Authors:** Agnieszka Golec de Zavala, Dorottya Lantos, Deborah Bowden

**Affiliations:** ^1^Department of Psychology, Goldsmiths, University of London London, UK; ^2^Department of Psychology, University of Social Sciences and Humanities Poznan, Poland; ^3^Instituto Universitário de Lisboa-Centro de Intervenção Social Lisbon, Portugal

**Keywords:** yoga, ‘power poses’, self-esteem, subjective sense of energy

## Abstract

Research on beneficial consequences of yoga focuses on the effects of yogic breathing and meditation. Less is known about the psychological effects of performing yoga postures. The present study investigated the effects of yoga poses on subjective sense of energy and self-esteem. The effects of yoga postures were compared to the effects of ‘power poses,’ which arguably increase the sense of power and self-confidence due to their association with interpersonal dominance (Carney et al., 2010). The study tested the novel prediction that yoga poses, which are not associated with interpersonal dominance but increase bodily energy, would increase the subjective feeling of energy and therefore increase self-esteem compared to ‘high power’ and ‘low power’ poses. A two factorial, between participants design was employed. Participants performed either two standing yoga poses with open front of the body (*n* = 19), two standing yoga poses with covered front of the body (*n* = 22), two expansive, high power poses (*n* = 21), or two constrictive, low power poses (*n* = 20) for 1-min each. The results showed that yoga poses in comparison to ‘power poses’ increased self-esteem. This effect was mediated by an increased subjective sense of energy and was observed when baseline trait self-esteem was controlled for. These results suggest that the effects of performing open, expansive body postures may be driven by processes other than the poses’ association with interpersonal power and dominance. This study demonstrates that positive effects of yoga practice can occur after performing yoga poses for only 2 min.

## Introduction

Recent research showed that momentarily assuming open and expansive body postures (‘power poses’) increased the self-reported sense of personal control and power (Carney et al., 2010). The authors attributed this effect to the meaning of interpersonal dominance that expansive body postures communicate: people who assume powerful poses look and feel in power. The reliability of the ‘power posing’ effects has been recently questioned (Ranehill et al., 2015; Simmons and Simonsohn, 2017). However, other research also suggests that momentarily performing open and expansive body poses may bring about changes in psychological states (e.g., Stepper and Strack, 1993; Briñol et al., 2009; Cuddy et al., 2012; Nair et al., 2015).

In addition, research on the practice of yoga – a non-competitive, physical exercise (asana) combined with breathing (pranayama) and meditation techniques (Sengupta, 2012) – indicates that practicing yoga is associated with improved psychological well-being (e.g., Shapiro and Cline, 2004; Kiecolt-Glaser et al., 2010) and positive self-esteem (Sethi et al., 2013). Practicing yoga postures improves vagal tone increasing bodily energetic resources (Wilhelm et al., 2004; Patil et al., 2013). This suggests that performing yoga postures may increase bodily energetic resources and the subjective sense of energy, and positively affect self-views. Thus, we propose that comparing the effects of ‘power poses’ to the effects of yoga asanas may suggest an alternative mechanism underlying the effect of body postures on psychological states. This mechanism may be related to the effects of the body alignment on the autonomous nervous system rather than to the meaning of interpersonal dominance, power or confidence associated with the posture.

Thus, in the present study we examined whether performing standing yoga poses with an erect spine and a lifted and open chest (with open vs. closed front of the body), in comparison to high and low ‘power poses’ (expansive vs. constrictive body postures), would affect two major contributors to psychological well-being and happiness: positive self-esteem – having a sense of self-worth and the subjective sense of energy – having energy and competence to accomplish goals (James, 1890; Ryan and Deci, 2001; Diener and Biswas-Diener, 2005; Lyubomirsky et al., 2006; Swann et al., 2007; Diener and Diener, 2009).

### Body Postures and Embodied Psychological States

Research shows that practicing yoga improves health and psychological well-being (Woodyard, 2011) and increases subjective feeling of energy, positive affect and self-esteem (Shapiro and Cline, 2004; Impett et al., 2006; Oken et al., 2006; Kiecolt-Glaser et al., 2010), but why this is the case is yet not well understood. Research points to beneficial psychological and neurological consequences of breathing and meditation techniques derived from yoga practice (Goyal et al., 2014). Less is known about the role of performing yoga poses (asanas) in improving psychological well-being. However, such effects can be inferred from existing literature on cognitive embodiment and on physiological effects of yoga poses.

Psychological embodiment research suggests that bodily postures and gestures in three dimensional spaces affect psychological states and attitudes. It has been argued that instrumental bodily experiences in one’s environment are incorporated into abstract thought. Manipulating bodily states can affect related, abstract thoughts and feelings (Niedenthal et al., 2005; Barsalou, 2008; Schubert and Semin, 2009; Meier et al., 2012). Directly related to personal empowerment, a study showed that people sitting in upright vs. slouched positions were more confident in the positive feedback they received and felt more proud of themselves (Stepper and Strack, 1993, Study 1). Participants who were sitting upright with a lifted chest vs. sitting in a slumped, chest collapsed position had more confidence in their thoughts (Briñol et al., 2009). Additionally, upright (vs. slumped) sitting increased arousal, positive mood and positive self-views just after the manipulation (Nair et al., 2015).

Recent research attributed the increase in the sense of power and personal control to the interpersonal meaning of the upright vs. slouched body postures. Studies have shown that people associate both erect, vertical (e.g., Schubert, 2005) and erect and open body postures (Carney et al., 2005; Hall et al., 2005) with power and dominance. Assuming high (erect, expansive, open) vs. low (slouched, constrictive body, arms covering upper body) ‘power poses’ for 2 min increased sense of power, confidence and personal control (Carney et al., 2010; Huang et al., 2011; Adam and Galinsky, 2012). It also increased participants’ testosterone levels – the hormone associated with dominance – and decreased afternoon cortisol levels, the hormone associated with responsiveness to stress. After performing the high power poses, participants also engaged in more risky gambling behavior (Carney et al., 2010, however, the latter two effects were not replicated (Ranehill et al., 2015; Garrison et al., 2016). In line with the associated meaning interpretation, Cesario and McDonald (2013) found the effects of expansive power poses on risk-taking behavior only when the poses were performed in a social context: when participants watched photographs of other people while holding the poses. In addition, assuming expansive poses elicited feelings of power in Western participants, but not in East Asian participants (Park et al., 2013). In some studies, performing ‘power poses’ did not influence psychological sense of power (e.g., Garrison et al., 2016).

It is not yet clear why the observed effects of ‘power posing’ are inconsistent. However, some research suggests that the confidence judgment (e.g., how easy or difficult it would be to climb a hill while standing at its feet) may depend on available bodily energetic resources (Schnall et al., 2010). When more of such resources are available people may feel more empowered and positive about themselves. Thus, it can be expected that performing body postures that increase bodily energetic resources may result in a greater subjective sense of energy and more positive self-views. It has been demonstrated that performing expansive yoga poses increased confidence in one’s own performance because it increased positive emotionality and the belief in one’s own ability and efficiency (Kok et al., 2013). According to BKS Iyengar (1965) – one of the best known teachers and propagators of yoga in the world – standing yoga poses that emphasize chest lifting and opening support deep breathing to the lungs’ capacity, which increases bodily energy, subjective feelings of energy and positive emotional state. In line with this proposition, research has linked deep breathing with increased vagal tone (stronger responses of the vagal nerve) and with physical and psychological well-being (Porges et al., 1994; Bowman et al., 1997; Pal et al., 2004; Kok et al., 2013). It has also been demonstrated that practicing yoga postures improves vagal tone (Powers and Howley, 2006; Goldstein et al., 2016) and increases heart rate variability indicative of a better vagal tone (Wilhelm et al., 2004; Patil et al., 2013).

Thus, performing expansive body postures may affect subjective feelings of energy and self-views, not because the poses convey the message of interpersonal dominance but because they increase bodily energetic resources. Yoga poses emphasize opening and lifting of the chest and stimulate the vagal nerve. They are not associated with competition or dominance^1^ but they are likely to increase physical energy and subjective sense of energy and empowerment. Thus, yoga poses offer a good comparison to ‘power poses’ for investigating whether the association with interpersonal dominance drives the effects of body poses on the subjective sense of energy and control. If performing yoga poses increases the subjective sense of energy and control when compared to performing ‘power poses,’ this may suggest that the effects of open body postures on psychological states may be real (which was recently questioned by one of the authors of the original research on ‘power posing’)^2^, but the underlying mechanisms of this effect may not be related to the meaning of dominance associated with the poses. ‘Power poses’ offer a meaningful comparison to test the immediate effects of momentarily performing standing yoga poses which have been neglected by research on psychological effects of yoga practice.

### The Effects of Yoga on Psychological Well-Being

Empirical findings indicate that practicing yoga benefits healthy individuals as well as those suffering from various physical or mental health problems (e.g., Jayasinghe, 2004; Innes et al., 2005; Innes and Vincent, 2007; Field, 2011; Roland et al., 2011). Practicing yoga improves mental health in the context of depressive disorders (Cramer et al., 2013b); anxiety disorders (Kirkwood et al., 2005); posttraumatic stress disorder (Mitchell et al., 2014); and schizophrenia (Cramer et al., 2013a). Yoga practice reduces stress (e.g., Michalsen et al., 2005) and performance anxiety (Khalsa et al., 2009). Practicing yoga is equally as effective as cognitive-behavioral therapy in stress management (Granath et al., 2006) and improving emotional health (Ross and Thomas, 2010). Specifically to psychological well-being, research conducted among patients prone to fatigue (e.g., recovering from cancer) reported lower fatigue after yoga practice interventions (Bower et al., 2005). Yoga practice reduces negative affect (e.g., Bower et al., 2012), increases positive affect and subjective feeling of energy (e.g., Shapiro and Cline, 2004; Kiecolt-Glaser et al., 2010) and positive self-esteem (Sethi et al., 2013).

However, studies on the psychological effects of yoga practice vary vastly in the duration of the examined yoga interventions, type of control group, and type of practice, and especially in whether or not they looked at physical practice in combination with breathing or meditation (Field, 2011). For example, studies that showed that yoga practice increases self-esteem looked at a 5-day yoga intervention combining exercising, breathing, meditation and teaching (Sethi et al., 2013) or 8 weeks of yoga practice (also combined with breathing, meditation and teaching) vs. physical exercise (combined with healthy lifestyle teaching based on modern Western knowledge) (Deshpande et al., 2009). Studies that showed that yoga practice improves satisfaction with life looked at the effects of 2 months of a yoga immersion program combining exercise, meditation and breathing (Impett et al., 2006) or a weekly 90-min yoga class and home practice over 6 months (Oken et al., 2006). Only a few studies have investigated the consequences of performing yoga poses (asana). Kiecolt-Glaser et al. (2010) showed that one session of practicing yoga poses in comparison to other physical exercise significantly increased positive affect. Shapiro and Cline (2004) showed that a yoga session containing mostly back bends (which expand the front of the body and chest area) improved affect and subjective feelings of energy in comparison to a session of forward bends (which cover the front of the body). In the present controlled, lab-based study, we separated the physical aspect of yoga practice – the performing of the asanas – as the independent variable. Unlike the previous studies, we examined the immediate effects of assuming standing yoga postures for 2 min only.

### Overview of the Present Study

Standing yoga poses, unlike expansive, high ‘power poses,’ emphasize the lift of the spine and the lift and openness of the chest rather than expansiveness of the body. Such positions allow full capacity of the lungs when breathing, which improves subjective sense of energy and mental performance (Birkel and Edgren, 2000). Certain standing yoga poses have arms crossed and covering the front of the body, making them comparable to constrictive, low ‘power poses.’ Thus, standing yoga poses can be compared to high, expansive and low, constrictive, ‘power poses.’ Yoga poses are devoid of associations with high or low power and dominance (they are not performed in order to assert either), but they are likely to create physiological changes that may affect psychological wellbeing. High ‘power poses,’ although erect and open, do not emphasize lift of the spine and expansion of the chest. Consequently, expansion of the chest and breathing to the lungs’ capacity may or may not occur when people are assuming those poses (which may explain the inconsistent results of ‘power posing’). Low ‘power poses’ emphasize slumping of the spine and decreasing the size of the chest.

In the present study, in a two factorial, between participants design, we compared standing yoga poses with or without arms crossed in front of the body to expansive, high and constrictive, low power poses, the effects of which on psychological empowerment have been demonstrated in previous studies (Carney et al., 2010; Ranehill et al., 2015). The study used a cover story to divert participants’ attention from the context of the body postures. Allegedly the study was examining the effect of fatigue on social cognition. If it is the meaning of dominance that the poses convey that affects psychological states, high power poses should increase the subjective feeling of energy and control and self-esteem in comparison to low power poses and yoga poses (both kinds, with the front of the body open and with arms crossed in front of the body, 3, -1, -1, -1 contrast). However, if the changes in psychological states are related to the effects of the poses on the autonomous nervous system, the yoga poses should increase the subjective sense of energy and control and self-esteem (both with the front of the body open and with arms crossed in front of the body) in comparison to both high and low power poses (a main effect).

## Materials and Methods

### Participants

Eighty-two undergraduate students in the UK (64 female, mean age = 22.99, *SD* = 6.02) participated in the study, which ostensibly examined the effects of fatigue on social perception. Participants were recruited via the University’s Research Participation Scheme. No exclusion criteria were applied. Based on effect sizes reported by Carney et al. (2015) and a similar study that used a different posing intervention (seated open chested or slouched poses, Nair et al., 2015, *r* = 0.37), based on power analysis by G^∗^Power, we aimed for a sample size of 76 participants to reach a power of 0.80 in a two factorial experimental study. Although we expected the main effect of yoga poses vs. power poses (which would suggest a sample size of 52), the literature may suggest an interaction between type of poses (yoga vs. power poses) and openness of the pose (open front of the body vs. covered front of the body). Thus, we used a more conservative sample estimation and collected more data in order to account for possible data loss. Data collection ceased on a predetermined date, and the data was not analyzed before this date.

Fifty-six participants were White, nine Black, six Asian and nine of mixed ethnicity, and one participant did not disclose their ethnicity. Park et al. (2013) reported that East Asian participants may perceive power poses differently to Western participants. However, excluding data from participants of ethnic minorities did not change the pattern of the results, thus the data of the whole sample was retained for the analyses.

### Design

A 2 (pose: power vs. yoga) × 2 (openness: open front of body vs. covered front of body) exclusively between-participants design was used. State self-esteem was measured as a dependent variable and the subjective sense of energy and control was measured as a mediator.

### Procedure

After giving their informed consent, participants responded to demographic questions (including a question regarding participants’ experience with physical exercise) and completed the Rosenberg Self-Esteem Questionnaire (Rosenberg, 1965) in order to assess their base-level self-esteem. They were then led into a lab cubicle to perform the body postures. The study closely followed the procedure of Carney et al. (2010) and Ranehill et al. (2015) using the same study materials and adding the yoga poses to the procedure. In line with the cover story that the study was investigating the link between fatigue and social perception, mock electrodes to measure fatigue were attached to participants’ wrists. Participants performed the poses alone in the lab cubicle listening to an audio recording with step-by-step instructions on how to assume the required body pose (**Table 1**). The order of poses was always the same. While performing the posture participants watched a slide show containing six photographs of people’s faces. Again in line with the cover story, participants were asked to form opinions about the people in the pictures (Carney et al., 2010).

**Table 1:** Verbatim instructions for poses played during the study.

	Instruction
Expansive Power Pose – Hands on hips	Stand between the ropes. Stand straight with your feet hip width apart. Have your feet parallel, toes slightly in. Straighten your legs. Bend your elbows. Take your hands to your hips, and take the elbows to the sides and slightly down. Lift your chest. Lift both sides of your waist equally and straighten your spine. Soften the tops of your shoulders down. Broaden your collarbones. Keep your chest broad and lifted. Have your chin parallel to the floor. Look at the computer screen.
Expansive Power Pose – Hands on table	Stand in front of the table in the middle of its narrower end. Step your right foot forward and slightly lean forward over the table, supporting your hands comfortably on the table. Have your hands shoulder width apart or slightly wider outside of the hooks. Lift and broaden your chest. Soften the tops of your shoulders sown toward your hips. Have your chin parallel to the floor. Look at the computer screen.
Constrictive Power Pose – Standing	Stand facing the computer, close to the wall behind you, with your back toward the fall. Stand straight with your feet hip width apart. Lean slightly back toward the wall. Bend your right leg slightly and cross you right shin over your left shin. Put your right foot on the floor on the outside of the left ankle. Bend your left arm. Cross it over your waist and put your left hand on the right side of your waist. Bend your right arm. Cross it over your chest, and get hold of your left middle upper arm with your right hand. Have your chin parallel to the floor. Look at the computer screen.
Constrictive Power Pose – Sitting	Take the chair and put it close to the wall in the place where you were standing. Sit on the chair with your feet on the floor hip width apart and your knees bent. Extended your arms in front of you and get hold of the edge of the chair in between you knees, or support your hands on your mid-thighs. Cross your right hand over your left hand. Lean slightly foreword. Have your chin parallel to the floor. Look at the computer screen.
Open front Yoga Pose – Tadasana/Samasthiti	Stand between the ropes. Stand straight with your feet hip width apart. Have your feet parallel, toes slightly in. Straighten your legs. Allow your arms to straighten down toward the floor alongside your body, palms facing your thighs. Actively stretch through your fingers toward the floor to straighten the arms. Lift your chest. Lift both sides of your waist equally and straighten your spine. Soften the tops of the shoulders. Keep your chest broad and lifted. Keep your chin parallel to the floor. Look at the computer screen.
Open front Yoga Pose – Urdhva Hastasana	Stand between the ropes. Stand straight with your feet hip width apart. Have your feet parallel, toes slightly in. Straighten your legs. Lift your chest. Lift both sides of your waist equally and straighten your spine. Soften the tops of your shoulders. Keep your chest broad and lifted. Without disturbing the position of your shoulders and chest, lift up your arms and take hold of the ropes. Walk your hands up on the ropes to straighten your arms up. Bring your elbows in. Lower the shoulders and lift the chest. Keep your chin parallel to the floor. Look at the computer screen.
Closed front Yoga Pose – Garudasana (left and right)	Stand between the ropes. Stand straight with your feet hip width apart. Have your feet parallel, toes slightly in. Straighten your legs. Lift your chest. Lift both sides of your waist equally and straighten your spine. Soften the tops of your shoulders. Keep your chest broad and lifted. Without disturbing the position of your shoulders and chest, lift your arms in front of you to the shoulder level, and take hold of the ropes. Hold the ropes extending the arms forward on the shoulder level. Turn the insides of your elbows toward the ceiling. Cross your right/left arm over your left/right arm. Bend the forearms up perpendicular to the floor. Lift the elbows to the level of your shoulders. Have your chin parallel to the floor. Look at the computer screen in between your forearms.


**FIGURE 1:**
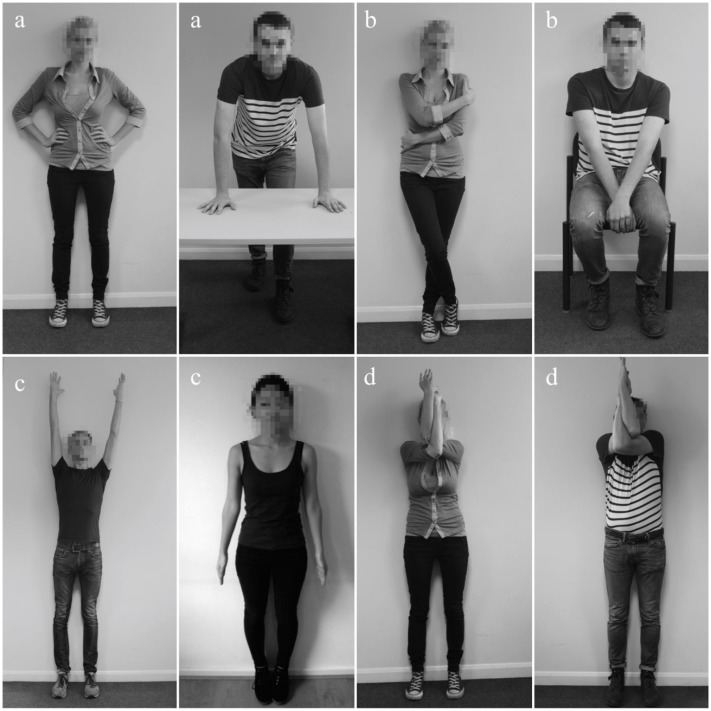
**The pictures of model poses used by the experimenter to make sure participants held the pose for 1 min to this standard:** **(a)** expansive power poses, **(b)** constrictive power poses, **(c)** open front of the body yoga poses, **(d)** closed front of the body yoga poses.

A computer-based random number generator was used to allocate participants to one of the four experimental conditions. Participants were asked to hold two standing yoga poses with open front of the body (*Tadasana, Urdhva Hastasana*, *n* = 19), or two standing yoga poses with arms crossed in front of the body (*Garudasana Right, Garudasana Left*, *n* = 22), or two expansive, high power poses (*Superman, Hands on the Table*, *n* = 21), or two constrictive, low power poses (*Standing, Seating*, *n* = 20), for 1-min each. The experimenter watched participants through a webcam to ensure that the poses were performed as instructed for 1 min and performed to the standards modeled in pictures presented in **Figure 1**. All participants complied with the instructions.

After each pose, participants completed a questionnaire which asked how comfortable, tiring, and difficult the pose was (answered on an 8-point Likert scale (0 = not at all, 7 = very much so). Those assessments did not correlate significantly with the dependent variable or the mediator (*ps* > 0.17) and were not analyzed further. Similarly, whether participants exercised infrequently, moderately or often did not affect the mediator or the dependent variable (*ps* > 0.70). Thus, participants’ level of experience with physical exercise was not analyzed further.

Once the electrodes were removed, participants responded to the questions assessing their subjective sense of energy, control, and power and completed the State Self-Esteem Scale (Heatherton and Polivy, 1991). Finally, participants were probed for guessing (no one guessed the true purpose of the experiment). Participants were rewarded with a course credit and a chocolate for completing the study.

#### Poses

The first expansive, high ‘power pose’ was the ‘superman pose’ (Tremain, 2013), a standing pose with hands resting on the hips and elbows pointing to the sides. The second expansive ‘power pose’ was the hands-on-table pose used by Carney et al. (2010). The legs-on-the-table ‘power pose’ used by those researchers was pre-tested for the present study together with all poses that were used in the study. However, it was perceived as significantly less dominant and powerful than other ‘high power’ poses. The constrictive, low ‘power poses’ were identical to those used by Carney et al. (2010): a standing pose with hands covering the body and the back slightly slouched, and a seated position, again with the participant’s back slightly slouched.

The standing yoga poses were chosen by a qualified Iyengar yoga instructor to be comparable to high and low power poses. The first open yoga pose was *tadasana*, or *mountain pose*: standing straight with the legs together and the spine and chest lifted. The second open yoga pose was *urdhva hastasana*: standing upright with the spine, chest and arms lifted. To make sure that this pose was not more physically straining than others, participants held ropes hanging from the ceiling for support. The yoga pose with arms crossed in front of the body was g*arudasana*, or *eagle pose*, where the arms are risen to be level with the shoulders and then crossed in front of the chest from the elbows upward. Participants first crossed their right arm over their left arm and then vice versa, again holding on to ropes for support. See **Figure 1** for an illustration of the standards for all poses and **Table 1** for verbatim of the instructions.

In order to ensure that the poses conveyed the meaning of high vs. low interpersonal dominance and self-confidence, 22 judges rated each pose on dominance (‘dominant’ and ‘powerful’) and self-confidence (‘confident’ and ‘self-assured’) dimensions using a 7-point Likert scale (“1” = “not at all” to “7” = “very much so”). A repeated measures ANOVA revealed that the mean of the dominance index ‘legs on table’ pose was lower than for the other two expansive, high ‘power poses,’ *F*(2,42) = 3.774, *p* = 0.031. The pairwise comparisons indicated a significant difference from the ‘hands on the table’ standing ‘power pose’, *p* = 0.004, but no significant differences between the other poses. Thus, this pose was not used in the Study.

The repeated measures ANOVA (with a Greenhouse–Geiser correction) for the dominance index indicated that (1) all open front of the body poses were seen as more dominant than all covered front of the body poses and (2) the open front of the body, high, ‘power poses’ were perceived as more dominant than the open front of the body yoga poses, *F*(2.06,43.24) = 30.88, *p* < 0.001, $\eta_{p}^{2}$ = 0.60 (Bonferroni correction, significant *p*s < 0.002). There was no significant difference between dominance attributed to covered, low ‘power poses’ and covered yoga poses, *p* = 1. A repeated measures ANOVA for the confidence index showed that (1) all open front of the body poses were seen as more self-confident than all covered front of the body poses and (2) the open, high ‘power poses’ were seen as significantly more self-confident than open front of the body yoga poses (Bonferroni correction, significant *p*s < 0.046), *F*(2.10,44.12) = 29.55, *p* < 0.001, $\eta_{p}^{2}$ = 0.59 (**Table 2**).

**Table 2:** Means and standard deviations for dominance and confidence indices in pre-test of poses.

	Dominance index	Confidence index
Yoga, closed front body	2.23 *(1.44)*	2.41 *(1.68)*
Yoga, open front body	4.28 *(1.61)*	4.51 *(1.60)*
(low) Power, closed front body	2.19 *(0.97)*	2.26 *(0.96)*
(high) Power, open front body	5.39 *(1.73)*	5.25 *(1.75)*


These results are in line with previous studies that indicate that people in erect, expansive body postures with open front of the body are attributed with more power than people in slouched and constrictive body postures (e.g., Carney et al., 2005; Hall et al., 2005). However, these results also show that people in open yoga poses are seen as less dominant and self-confident than people assuming ‘high power poses.’

#### Measures

##### Trait self-esteem

Participants’ baseline trait self-esteem was measured by the 10 item Rosenberg Self-Esteem Scale (Rosenberg, 1965). Participants responded on a scale from 0 (strongly disagree) to 3 (strongly agree) to items such as “*I feel that I have a number of good qualities*” or “*I certainly feel useless at times*” (reversely coded) (*α* = 0.88, *M* = 1.98, *SD* = 0.5).

##### State self-esteem

State self-esteem was assessed by the 20 item State Self-Esteem Scale (Heatherton and Polivy, 1991). Participants responded on a scale from 1 (not at all) to 5 (extremely) to items pertaining to how they felt about themselves in the present moment: “*I feel confident about my abilities” or “I feel that others respect and admire me,*” (*α* = 0.89, *M* = 3.5, *SD* = 0.62).

##### Subjective energy

Subjective feelings of energy were measured by six questions asking participants how they felt at the present moment: “*I feel in control,” “I feel powerful,” “I feel energetic,*” and “*I feel empowered.*” Participants answered on a scale from 1 (not at all) to 5 (extremely), (α = .82, *M* = 2.8, *SD* = 0.79). The Principal Components Factor Analysis performed on these items revealed one factor solution with the factor loading of 2.35 accounting for 58.83% of variance. Item loadings ranged from 0.62 to 0.88.

## Results

**FIGURE 2:**
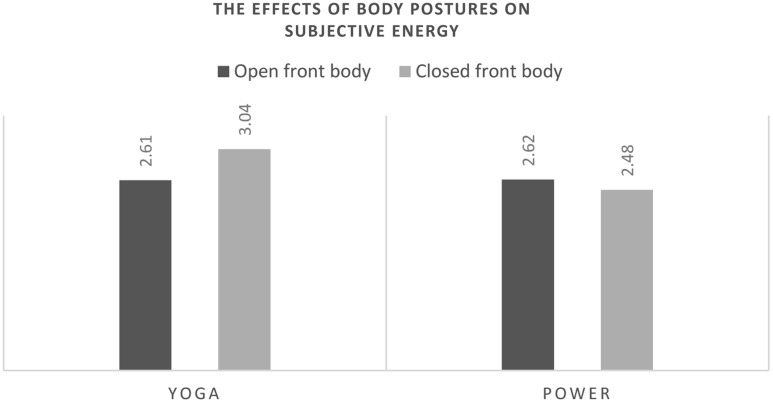
**The effects of the body postures on the subjective sense of energy.** The significant difference is between yoga vs. power postures.

Subjective sense of energy and trait and state self-esteem were positively correlated across research conditions (**Table 3**). In order to test the hypothesis that performing yoga poses should increase state self-esteem through increasing subjective sense of energy, we first tested whether performing the yoga vs. ‘power’ poses affected the subjective sense of energy. We performed a 2 (pose type: yoga vs. power) by 2 (openness: expansive, open front of body vs. constrictive, covered front of body) GLM expecting a main effect of yoga poses. The analyses included trait self-esteem as a covariate. Trait self-esteem was found to correlate with state self-esteem and subjective sense of energy. This is in accordance with previous studies, which have shown trait self-esteem to be related to psychological sense of power (e.g., Lecomte et al., 1997, 1999; Corrigan et al., 1999). Thus, controlling the variance due to trait self-esteem in subjective sense of energy allowed us to make a clearer assessment of the variance due to the experimental manipulation of body postures^3^. This analysis produced a non-significant main effect of openness of the pose, *F*(1,77) = 0.06, *p* = 0.81, $\eta_{p}^{2}$ = 0.001, and a significant main effect of pose type, *F*(1,77) = 6.18, *p* = 0.015, $\eta_{p}^{2}$ = 0.07. The interaction of pose type and openness was non-significant, *F*(1,77) = 0.01, *p* = 0.94, $\eta_{p}^{2}$ < 0.001. The mean estimates presented in **Table 4** indicate that the subjective sense of energy was higher after performing all yoga poses in comparison to all ‘power poses.’ This effect was not driven by decreased sense of energy after performing ‘low power poses,’ as the difference in the means of the sense of energy between ‘high’ and ‘low power poses’ was negligible (**Table 4** and **Figure 2**).

**Table 3:** Correlations between variables.

Measure	1	2	3
(1) Trait self-esteem	–		
(2) State self-esteem	0.71^∗∗^	–	
(3) Subjective energy	0.53^∗∗^	0.66^∗∗^	–


*^∗∗^*p* < 0.001.*

**Table 4:** Means (and standard deviations) of the observed effects.

		Pose type		
			
DV	Openness	Yoga	Power	Total
Subjective energy	Open front body	2.61 *(0.78)*	2.62 *(0.75)*	2.61 (0.76)
	Closed front body	3.04 *(0.79)*	2.48 *(0.90)*	2.77 (0.88)
	Total	2.82 (0.81)	2.55 (0.82)	


**FIGURE 3:**
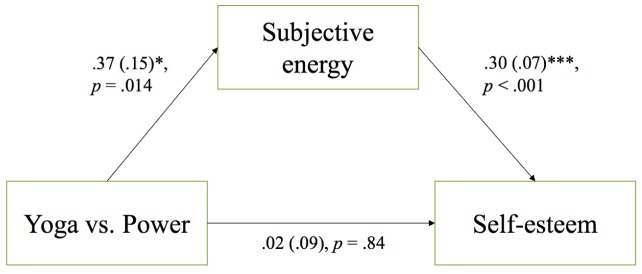
**Standardized regression coefficients, standard errors, and probability values for the relationship between pose and state self-esteem, as mediated by the subjective sense of energy.** ^∗^*p* < 0.05, ^∗∗∗^*p* < 0.001.

Next, we performed a mediation analysis using a multiple regression approach and Model 4 in PROCESS macro for SPSS (Hayes, 2013). Pose type (dummy coded 0 for power poses and 1 for yoga poses) was entered into the model as the predictor, state self-esteem as the outcome variable, and subjective sense of energy as the mediator. Trait self-esteem and openness of the pose were entered as covariates. The analyses used 1000 bootstrap samples. The overall model was significant, *R^2^* = 0.62, *F*(4,77) = 30.77, *p* < 0.001. The indirect effect of pose type on state self-esteem mediated through subjective sense of energy was significant with a point estimate of 0.11, and 95% CI [0.03, 0.27] (**Figure 3**). The results showed the same pattern when the analysis was performed without covariates. The indirect effect of pose type on state self-esteem mediated via subjective sense of energy was significant with a point estimate of 0.17, and 95% CI [0.01, 0.39].

## Discussion

The results of the present study suggest that momentarily performing standing yoga poses (with open or covered front of the body) in comparison to ‘high’ and ‘low power poses’ (expansive and constrictive body postures tested in previous studies) improved state self-esteem through increasing the subjective sense of energy and empowerment: feeling in control, in power, energetic and empowered. This effect was particularly pronounced when baseline trait self-esteem was controlled for. This indicates that the effect of the yoga poses on state self-esteem was independent of participants’ trait-like self-view and occurred over and above the effect of participants’ typical self-views.

The present results are in line with previous research pointing to positive consequences of yoga practice on psychological well-being. It has previously been shown that regular practice of yoga (including breathing and meditation) reduced fatigue (e.g., Bower et al., 2012), increased subjective feelings of energy (Shapiro and Cline, 2004), promoted empowerment (Ojha, 2013; Vyas-Doorgapersad and Surujlal, 2014), and increased self-esteem (e.g., Sethi et al., 2013). The results of the present study go beyond such findings and indicate that the increase in the subjective sense of energy and control and self-esteem can be experienced immediately after momentarily performing standing yoga postures. In previous research, Shapiro and Cline (2004) showed that 90 min sessions of back-bending and standing poses improved positive affect and subjective feelings of energy. The present research indicates that 2 min of standing with an erect spine and lifted chest increased the subjective sense of energy and self-esteem in comparison to four other open and constrictive, standing and seated body postures. While Shapiro and Cline (2004) noted that the immediate positive effects of yoga may be apparent for about 2 h following a yoga class, the duration of the immediate effects of only briefly assuming yoga poses had not been examined.

The current findings suggest that momentarily performing standing yoga poses (in comparison to other standing and seated body postures) increases subjective sense of energy which in turn increases positive self-view. Although further studies are needed to investigate the physiological correlates of this effect, the present findings are in line with previous studies suggesting that yoga practice stimulates the vagal nerve (Wilhelm et al., 2004; Powers and Howley, 2006; Patil et al., 2013; Goldstein et al., 2016), whose improved tone is related to increased subjective feelings of energy and well-being (Kok et al., 2013) and higher self-esteem (Martens et al., 2008). Seen from this perspective, the present results are also in line with studies demonstrating that even short interventions may improve the vagal tone and bring associated positive outcomes (Mäkinen et al., 2008; Kok and Fredrickson, 2010; Kok et al., 2013). The present findings indicating that even momentarily performing yoga poses significantly increased the subjective sense of energy and control and increased the self-esteem may hold important implications for those who practice yoga or work on creating yoga-based intervention programs for individuals suffering from mental or physical disorders and also on programs aiming at improving the psychological well-being of healthy populations. The present findings suggest that even short practice of simplified yoga poses can have positive effects on well-being and self-views.

The results of the current study are not consistent with the proposition that the meaning of body postures – their association with interpersonal dominance – drives their effects on the subjective sense of empowerment and self-esteem. The pre-test of the poses for the British study indicated that people performing expansive ‘power poses’ were seen as more powerful and dominant than those performing ‘low power,’ constrictive poses and those performing all yoga poses (with the chest open and with the arms crossed in front of the body). However, the increase in the subjective sense of energy and self-esteem was not observed in the ‘high power’ postures condition in comparison to all other conditions. If the effects of body postures were driven by the fact that all expansive and open body postures communicate self-confidence or dominance (which was not confirmed by the pre-test), a main effect for the openness of the pose would have been expected (or the specific contrast of high power poses being the most effective). However, no such effects were observed. The increases in the subjective sense of energy and control and self-esteem were observed when all yoga poses were performed. This suggests that another mechanism drives the effect of open body postures on the subjective sense of empowerment and self-esteem, before the effect of the meaning of the pose can be observed.

There was also no noticeable drop in the subjective sense of energy or state self-esteem in the ‘low power poses’ condition relative to the ‘high power poses’ condition. This suggests that the differences between yoga and power poses were unlikely to be driven by ‘low power,’ constrictive poses decreasing the subjective sense of energy or state self-esteem. The lack of observed differences between constrictive and expansive yoga poses might be due to the fact that even the constrictive yoga pose – crossing the arms across the front of the body – may still expand the chest and holds the spine erect. This pose is not as constrictive and back-rounding as those used in previous research.

The study is not without limitations that render cautious interpretation of its preliminary results. Firstly, it tested the effects of only four yoga poses. It has been estimated that there are roughly 840,000 *asana*s, approximately 84 of which are used regularly in yoga practice (Jayasinghe, 2004). The presented poses were chosen to be as similar to the ‘power poses’ as possible and easy to perform. It is possible that some yoga poses might affect psychological states differently, and further research is warranted to investigate this. The study also did not control for the possibility that yoga poses may have benefits due to placebo, brought about by positive expectations associated with yoga. However, as participants did not know the real purpose and aims of the experiment and were not informed they were performing yoga poses, it is likely that the observed effects resulted from the poses alone.

The study did not control for the level of experience in yoga practice which might have affected how participants performed the poses. However, the study used the random assignment to experimental conditions which should prevent the confounding effect of yoga practice. In addition, participants’ level of experience with physical exercise was not related to the state self-esteem or the subjective sense of energy which increases our confidence in the results pointing to the momentary effects of yoga postures.

It is important to note the variations in the way the yoga poses were performed. Participants held ropes in the standing poses and performed the poses in front of the computer screen. The former variation was added in order to make the yoga poses comparable to ‘power poses’ in the level of difficulty as unassisted *urdhva hastasana* and *garudasana* require muscle strength and flexibility which are not crucial to perform the ‘power poses.’ Indeed there were no differences between the poses in the perceived level of difficulty or discomfort. Yoga poses, especially as instructed by BKS Iyengar, are often performed with the use of props in order to emphasize alignment over the individual skill or fitness. Thus, the use of props should have not distracted but rather facilitated the proper alignment in the pose. Poses were performed in front of the computer because the study followed closely the methodology of the ‘power posing’ studies. Arguably, yoga poses might have not been typically performed in the presence of screens; however, nowadays it is not uncommon for yoga to be practiced in gyms. Whether the effects of the practice are undermined by this context remains an empirical question.

Finally, despite the fact that the sample size was appropriate according to the estimation performed *a priori* based on the existing literature, replication of the study would be desirable. Although the sample size was comparable to those used in previous studies investigating the effects of body postures on psychological well-being (Nair et al., 2015) and almost twice as large as the sample in the original study investigating the effects of power posing (Carney et al., 2010), recent analyses suggest that effect sizes from ‘power posing’ studies – on which the sample estimation for the present study was based – may not be reliable (Simmons and Simonsohn, 2017).

Relatedly, the findings of the study do not accord with those reported by Carney et al. (2010), Huang et al. (2011), Tremain (2013), or Ranehill et al. (2015), despite following the procedure in regards to inducing ‘high’ and ‘low’ power postures in great detail^4^. More specifically, participants did not feel more ‘in power’ and ‘in control’ after performing the ‘high’ vs. ‘low power poses.’ However, unlike the previous studies, the present study was conducted in the UK, so the inconsistency in findings may be due to the cultural variation in the interpretation of body postures (Remland et al., 1995; Kleinsmith et al., 2006; Park et al., 2013). In addition, the sample in the present study was quite ethnically diverse, with 69.5% of participants identifying as White, and the rest of the sample made up of participants from various ethnic minorities. Previous studies in other domains of embodied cognition suggest that some embodied effects may be dependent on specific culturally transmitted metaphorical associations and differ from culture to culture (Lee et al., 2015). However, the meaning of dominance and confidence associated with the poses was pre-tested on a UK sample prior to the study, and the results indicate that people in ‘high power poses’ were seen as more dominant and powerful than people in any other body poses. This suggests that British participants, irrespective of their ethnicity, saw the high power poses similarly to participants of the previous studies.

## Conclusion

The present study demonstrated that momentarily performing four standing yoga poses in comparison to ‘power poses’ (expansive and constrictive body postures expressing high vs. low interpersonal dominance) improved state self-esteem. This effect was independent of trait self-esteem and driven by an increase in the subjective sense of being energetic, empowered and in control. This increase occurred in comparison to ‘power poses’ argued to increase the subjective sense of empowerment. The findings suggest that the effects of body postures on the subjective sense of empowerment are most likely driven by a different mechanism than their association with interpersonal dominance. Although this results are preliminary, they suggest that yoga poses can positively affect psychological states. The results have potential applicability in interventions for improving psychological well-being. They also point to the need for improvements in our theorizing regarding the mechanisms driving the effects of open body poses on psychological states.

## Ethics Statement

The study was carried out in accordance with the recommendations of the British Psychological Society. Participants were asked to sign an informed consent form before taking part in the study. All participants gave written informed consent in accordance with the Declaration of Helsinki. The protocol was approved by the Ethical Committee for Psychological Research at the Department of Psychology at Goldsmiths, University of London. The Committee examined the detailed description of procedure and measurements used in the study. Participants were informed about the study’s procedure but a cover story was used as to the true purpose of the study. The cover story informed participants that the research would examine the link between fatigue and social perception. Participants were fully debriefed upon completion of the study. They were informed that their data would be used for scientific purposes only and that their participation was anonymous and their data could be withdrawn at any stage of conducting, analyzing and presenting the data.

## Author Contributions

AGdZ produced the research question, theoretical rationale and hypothesis, designed the study, oversaw the process of data collection, conducted the statistical analyses and collaborated on writing up the manuscript. She was responsible for presenting the main line of argument. DL prepared and developed the materials for the study, collaborated in data collection, prepared the first draft of the manuscript. She collaborated on writing up the manuscript. She was responsible for presenting the literature review on power posing and psychological effects of yoga and description of research method and results. DB collaborated on study design and preparation of the methods, she oversaw the data collection and helped collect data. She collaborated on writing up the manuscript. She oversaw the final editing and presentation of the manuscript.

## Conflict of Interest Statement

The authors declare that the research was conducted in the absence of any commercial or financial relationships that could be construed as a potential conflict of interest.

## Footnotes

1 It can be argued that the names of some yoga poses such as ‘warrior’ pose or ‘child’s’ pose suggest positions of low vs. high power. In order to ensure that the meaning that could be associated with the pose did not affect the subjective feeling of energy and self-esteem we pretested the meaning associated with each pose. The pretest (below) showed that dominance and confidence was attributed to ‘high power poses’ more than to open front of the body yoga poses and to all covered front of the body poses.

2 http://faculty.haas.berkeley.edu/dana_carney/pdf_My%20position%20on%20power%20poses.pdf

3 When the trait self-esteem was not included as a covariate the effect of openness of the pose was not significant, *F*(1,78) = 0.07, *p* = 0.82, $\eta_{p}^{2}$ = 0.002, and the main effect of pose type was marginally significant, *F*(1,78) = 3.29, *p* = 0.07, $\eta_{p}^{2}$ = 0.04. The interaction of pose type and expansiveness was non-significant, *F*(1,78) = 1.31, *p* = 0.26, $\eta_{p}^{2}$ = 0.02.

4 The only difference in the procedure of the present study was one of the high ‘power poses’ participants were asked to perform. Instead of an expansive sitting pose with arms bent behind the head and feet crossed on the table, the present study used an expansive standing pose with hands on the hips and elbows bent to the side. This pose was used in previous research showing that it increases the sense of power (Tremain, 2013). The poses used in the power constrictive condition were identical to those used in previous research.
