# Corrigendum: Yoga Poses Increase Subjective Energy and State Self-Esteem in Comparison to ‘Power Poses'

**DOI:** 10.3389/fpsyg.2018.00149

**Published:** 2018-02-09

**Authors:** Agnieszka Golec de Zavala, Dorottya Lantos, Deborah Bowden

**Affiliations:** ^1^Department of Psychology, Goldsmiths, University of London, London, United Kingdom; ^2^Department of Psychology, University of Social Sciences and Humanities, Poznan, Poland; ^3^Instituto Universitário de Lisboa-Centro de Intervenção Social, Lisbon, Portugal

**Keywords:** yoga, ‘power poses', self-esteem, subjective sense of energy

In the original article, there was a mistake in Figure 3. The statistics illustrating the direct effect of pose type on subjective energy are missing from the figure. The corrected Figure 3 appears below. The authors apologize for this error and state that this does not change the scientific conclusions of the article in any way.

**Figure 3:**
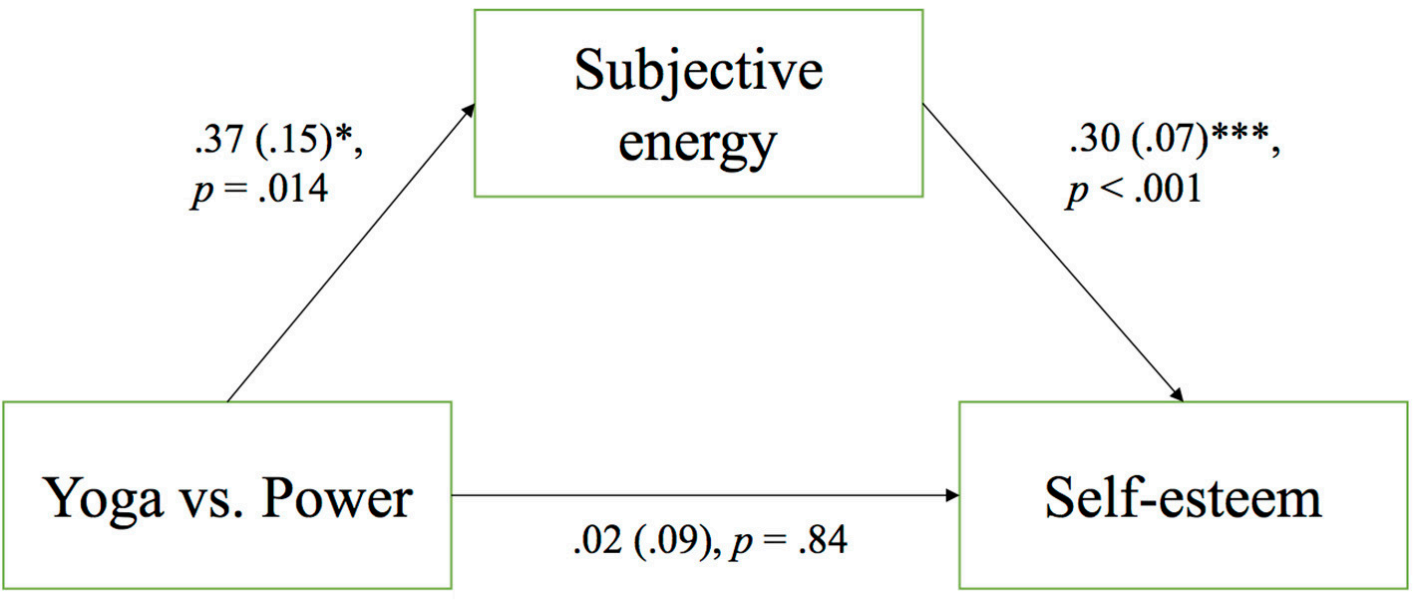
Standardized regression coefficients, standard errors, and probability values for the relationship between pose and state self-esteem, as mediated by the subjective sense of energy. *p < 0.05, ***p < 0.001.

The original article has been updated.

## Conflict of interest statement

The authors declare that the research was conducted in the absence of any commercial or financial relationships that could be construed as a potential conflict of interest.

